# Normal caloric intake with high-fat diet induces metabolic dysfunction-associated steatotic liver disease and dyslipidemia without obesity in rats

**DOI:** 10.1038/s41598-024-74193-y

**Published:** 2024-10-01

**Authors:** Mateusz Szudzik, Tomasz Hutsch, Dawid Chabowski, Mikołaj Zajdel, Marcin Ufnal

**Affiliations:** 1 Laboratory of Centre for Preclinical Research, Department of Experimental Physiology and Pathophysiology, Medical University of Warsaw, Pawińskiego 3c Street, Warsaw, Poland; 2 Department of Pathology and Veterinary Diagnostics, Institute of Veterinary Medicine, Warsaw University of Life Sciences (WULS-SGGW), Nowoursynowska 159c, Warsaw, Poland; 3 Veterinary Diagnostic Laboratory ALAB bioscience, 22/30 Stępińska Street, Warsaw, Poland

**Keywords:** Diet, MASLD risk factors, PCSK9, Liver steatosis, Cholesterol metabolism, Low-density lipoprotein, Molecular biology, Physiology, Diseases, Gastroenterology, Medical research, Risk factors

## Abstract

Excessive caloric intake and obesity due to high-fat (HFD) and high-disaccharide (HDD) diets have been recognized as major contributing factors to dyslipidemia and metabolic dysfunction-associated steatotic liver disease (MASLD). However, the effect of HFD and HDD without excessive caloric intake is obscure. The aim of the study was to evaluate the effect of physiological caloric intake delivered through HFD and HDD on liver and lipid profiles. The study was performed on 6-week-old male and female (50/50%) Sprague Dawley rats, receiving either a standard (controls, *n* = 16), HFD (*n* = 14) or HDD (*n* = 14) chow. All groups received the same, standard daily calorie rations, titrated weekly to the age of growing rats, for 12 weeks. A panel of metabolic in vivo measurement were performed, followed by histological, biochemical and molecular biology assays on tissues harvested from sacrificed rats. There was no significant difference between the groups in body weight. In contrast to controls, HFD and HDD groups showed metabolic dysfunction-associated steatohepatitis (MASH) characterized by liver steatosis, inflammation, ballooning of hepatocytes and fibrosis. These changes were more pronounced in the HFD than in the HDD group. The HFD group showed significantly higher serum LDL than controls or HDD rats. Furthermore, the HFD group had higher liver protein levels of low-density lipoprotein receptor (LDLR) but lower plasma levels of proprotein convertase subtilisin/kexin type 9 (PCSK9) than the controls or HDD group. There were no differences between sexes in evaluated parameters. The excessive caloric intake and obesity are not prerequisites for the development of MASH and dyslipidemia in rats. The liver changes induced by the HFD and HDD diets exhibit differences in severity, as well as in the expression patterns of LDLR and PCSK9. Notably, these effects are independent of the sex of the rats.

**Supplementary Information:**

The online version contains supplementary material available at 10.1038/s41598-024-74193-y.

## Introduction

According to recent changes in nomenclature, Non-Alcoholic Fatty Liver Disease (NAFLD) and Non-Alcoholic Steatohepatitis (NASH) are now referred to as Metabolic Dysfunction-Associated Steatotic Liver Disease (MASLD) and Metabolic Dysfunction-Associated Steatohepatitis (MASH), respectively^1^. This manuscript will adhere to the new nomenclature.

MASLD is estimated to affect almost two billion people globally^2,3^. Liver-specific mortality is associated with MASH, the most aggressive type of MASLD, which has a prevalence of up to 6%^2^. In general, the incidence and severity of MASLD are higher in men than in women, probably due to the protective effect of estrogen. However, the incidence of MASLD in women has been reported to increase after menopause^5^.

Recent decades have registered rapid growth of MASLD diagnoses, but minimal progress has been made in prevention and pharmacological treatment. Wide gaps in the knowledge of pathological processes of MASLD hinder the development of new and more effective preventive and therapeutic measures^6,7^. The accumulation of fat in liver (hepatic steatosis) resulting from causes other than excessive alcohol consumption, pharmacotherapy or other diseases is characteristic of MASLD. Based on the histological picture MASLD may be divided into the fatty liver and MASH. Fatty liver is hepatic steatosis with no evidence of hepatocellular injury presenting as hepatocyte ballooning, whereas MASH is characterized by hepatic steatosis, inflammation with hepatocyte ballooning with or without fibrosis^8^.

MASLD is thought to be caused by multiple factors, including insulin resistance, genetic factors, obesity and an unbalanced diet^9^. Diet is indicated as a major factor in the development of MASLD^10^. In particular, the so-called western diet, characterized by overconsumption of highly processed food containing over-refined sugars, refined and saturated fats and animal protein with the simultaneous restriction of the consumption of plant-based products, seems to be causally related to MASLD^11,12^.

Excessive caloric intake, together with high-fat (HFD) and high-disaccharide diets (HDD), have been shown to produce metabolic disorders, including MASLD across its entire spectrum in laboratory animals^13–15^ under ad libitum food settings. However, the effect of HFD and HDD diets without excessive caloric intake and/or obesity has not been elucidated.

In this study, we evaluated the effect of HFD and HDD diets under conditions of restricted food intake to provide normal/physiological energy intake on the liver and lipid metabolism, including low-density lipoprotein receptor (LDLR) and proprotein convertase subtilisin/kexin type 9 (PCSK9) expression in male and female rats.

## Results

### HFD, HDD and control groups showed similar weight gain

There were no significant differences between the groups in body weight, energy intake and other evaluated parameters before the start of the experimental intervention (Table 1).

**Table 1:** Metabolic parameters at the beginning and end of the study calculated per 100 g of body weight (BW). Sprague Dawley rats maintained on control, high- disaccharide (HDD) and high-fat (HFD) diets for 12 weeks.

		Controls	HDD	HFD	ANOVA
Start	Initial body weight (g)	185.75 ± 7.34	182.26 ± 6.29	182.30 ± 5.69	*P* = 0.83
	Energy intake (kcal/day/100 g BW)	37.46 ± 0.13	38.13 ± 0.17	38.24 ± 0.15	*P* = 0.90
	Water Intake g/100 g BW	13.63 ± 0.60	13.15 ± 0.56	14.48 ± 0.66	*P* = 0.31
	Urine output g/100 g BW	3.30 ± 0.27	3.22 ± 0.22	3.69 ± 0.40	*P* = 0.63
	Stool output g/100 g BW	5.14 ± 0.41	5.88 ± 0.38	5.59 ± 0.27	*P* = 0.24
End	Final body weight (g)	391.07 ± 26.07	384.59 ± 25.62	368.40 ± 23.02	*P* = 0.67
	Energy intake (kcal/day/100 g BW)	16.17 ± 0.09	16.21 ± 0.09	16.85 ± 0.08	*P* = 0.54
	Water Intake g/100 g BW	6.21 ± 0.76	3.87* ± 0.31	5.29 ± 0.65	*P* = 0.03
	Urine output g/100 g BW	2.89 ± 0.42	1.78* ± 0.23	2.16 ± 0.31	*P* = 0.01
	Stool output g/100 g BW	0.85 ± 0.13	0.47* ± 0.04	0.97## ± 0.12	*P* = 0.001

* *P* < 0.05, **P < 0.01 vs. controls; # *P* < 0.05, ##*P* < 0.01 HDD vs. HFD Means ± SE are presented.

Males had greater body weight than females at the end of the experiment but there were no differences between groups within the same sex (Supplementary Table 3). During the study, the body weight gain was as expected for rats at this age^16^.

## HFD had significantly higher LDL levels than the controls or HDD

There were significant differences between the groups in lipid profile. HFD rats had significantly higher LDL plasma levels than the controls or HDD rats. The HDD group had a significantly higher total cholesterol (*P* < 0.05) and triglycerides (*P* < 0.05) compared to controls and significantly higher triglycerides (*P* < 0.01) than HFD rats. At the same time, no differences were found in low-density lipoprotein (LDL) and high-density lipoprotein (HDL) between the HDD group and the controls (Table 2).

**Table 2:** Lipid profile, glucose, serum PCSK9 level, sodium, potassium and aminotransferases in Sprague Dawley rats maintained on control, high-disaccharide (HDD) or high-fat (HFD) diets for 12 weeks.

	Controls	HDD	HFD	ANOVA
Total cholesterol (mg/dL)	57.93 ± 1.74	66.57* ± 3.01	63.07 ± 2.21	*P* = 0.03
LDL (mg/dL)	8.48 ± 0.41	8.67 ± 0.86	11.35*# ± 0.77	*P* = 0.01
HDL (mg/dL)	46.26 ± 1.63	46.01 ± 3.16	51.57 ± 2.28	*P* = 0.22
Triglycerides (mg/dL)	132.53 ± 11.17	177.86* ± 17.04	115.64## ± 8.28	*P* = 0.001
Glucose (mg/dL)	213.86 ± 10.57	238.58 ± 9.71	219.36 ± 12.88	*P* = 0.22
PCSK9 (ng/ul)	152.52 ± 20.55	189.70## ± 5.80	105.66* ± 10.93	*P* = 0.02
Sodium (mmol/l)	138.75 ± 0.91	138.07 ± 1.04	138.54 ± 0.59	*P* = 0.85
Potassium (mmol/l)	4.38 ± 0.09	4.60 ± 0.13	4.85 ± 0.36	*P* = 0.30
ALT (U/l)	22.55 ± 7.86	20.50 ± 8.36	21.00 ± 10.61	*P* = 0.97
AST (U/l)	137.90 ± 43.93037	149.14 ± 38.04133	144.16 ± 96.91113	*P* = 0.58
Creatinine clearance (ml/min)	1.15 ± 0.12	0.86 ± 0.07	1.06 ± 0.11	*P* = 0.13

*P < 0.05, **P < 0.01 vs. controls; # *P* < 0.05, ##*P* < 0.01 HDD vs. HFD Means ± SE are presented.

There were no significant differences in serum glucose, sodium, potassium or aminotransferases levels between the experimental groups and between males and females (Supplementary Table 4).

## HFD but not controls developed MASH-like phenotype

**Fig. 1:**
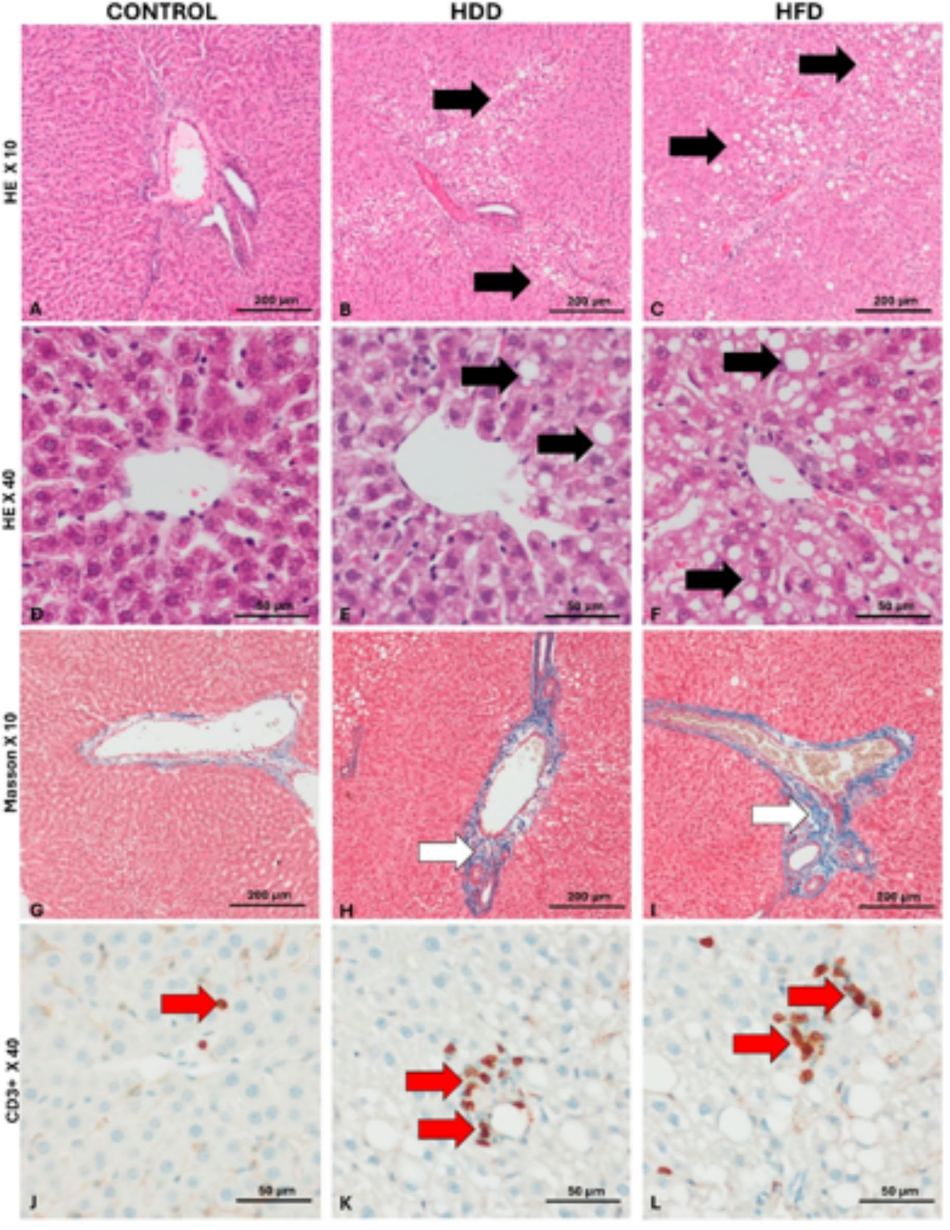
Histopathological images of the liver. A-F - hematoxylin-eosin staining; G-I - Trichrom Masson staining; J-L - anti-CD3 immunohistochemical staining. Black arrows - foci of hepatocyte steatosis, White arrows- hyperplasia of connective tissue around the veins in the portal spaces, Red arrows- lymphocytes/foci of lymphocytes in the liver parenchyma.

Overall, no significant histopathological changes were observed in the control group. The MASH-like phenotype was observed in 57% of HFD and 27% of HDD rats. There was a significant difference in the degree of hepatocyte steatosis between the control group and HFD rats. In addition, the HDD group showed greater infiltration of T lymphocytes in the liver parenchyma compared to controls. Moreover, rats from the HDD and HFD group showed a significantly greater number of ballooning of hepatocytes and greater fibrosis around the veins in the liver portal area (Fig. 1).

The observed pathological processes were most pronounced in the HFD group. Degenerative and fatty changes were observed mainly in the central part of the lobules. Minimal extramedullary hematopoiesis in the liver was found in the HDD group (Fig. 1).

The scoring analysis demonstrates that changes varied in severity between the studied groups (Table 3). The observed changes were significant in the HFD group and moderate in the HDD group. In the control group, the level of changes was minimal to slight. Female rats show more pronounced changes in liver steatosis compared to males (Supplementary Table 5).

**Table 3:** Results of histopathological scalar assessments of the liver for the three study groups.

Histopathological parameters			Control (*n* = 15)	HDD (*n* = 14)	HFD (*n* = 14)
Steatosis scoring	**SCORE**	0 I II III	11 2 2 0	7 3 4 0	5 2 3 4
	*(Sum; Median [QD])*		6; 0 [0.25]	11; 0.5 [0.825]	22; 1.5 [1.25]*
Inflammation scoring	**SCORE**	0 I II III	10 5 0 0	2 6 5 1	3 8 2 1
	*(Sum; Median [QD])*		8; 0 [0.5]	19; 1 [0.5]*	15; 1 [0.0]
Ballooning hepatocytes scoring	**SCORE**	0 I II	14 1 0	4 6 4	2 11 1
	*(Sum; Median [QD])*		1; 0 [0.0]	14; 1 [0.75]*	13; 1 [0.0]*
Fibrosis scoring	**SCORE**	0 I II III	13 2 0 0	12 2 0 0	9 5 0 0
	*(Sum; Median [QD])*		2; 0 [0.0]	2; 0 [0.0]	5; 0 [0.5]
***% of individuals with MASLD or MASH in the group***			13.33%	50%	64.28%
***% of individuals with MASLD in the group***			13.33%	21.43%	7.14%
***% of individuals with MASH in the group***			0.00%	28.57%	57.14%

For each histopathological criterion the sum of scoring points and their median with quarter deviation (QD) were calculated.* *p* < 0.05 vs. Control in non-parametric Kruskal-Wallis test for multiple comparisons.

## HFD showed higher liver LDLR and lower PCSK9 blood level than controls

**Fig. 2:**
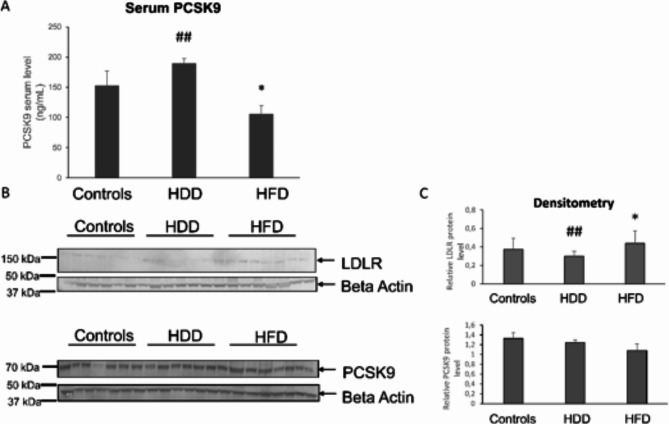
Serum and hepatic PCSK9/LDLR proteins levels. A PCSK9 serum levels in Controls, HDD and HFD rats; B Immunoblots of indicated proteins in the liver of Controls, HDD and HFD rats; C Densitometry quantification of each protein relative to beta actin; *P < 0.05 vs. Controls, # *P* < 0.05, ##*P* < 0.01 HFD vs. HDD, Means ± SE are presented for *n* = 42.

In general, there were significant differences in the protein levels between the groups (Fig. 2A-C). There was a higher level of LDLr protein in liver tissue homogenates from HFD rats compared to the other two groups. Liver PCSK9 protein levels did not differ significantly between the groups (Fig. 2B, C). HFD rats presented a significantly lower level of PCSK9 in serum compared to HDD and controls (Fig. 2A). No changes were found between males and females rats in serum PCSK9 (Supplementary Figs. 1,2).

## Discussion

The novel finding of the present study is that a physiological caloric intake, when provided through a high-fat or high-disaccharide diet, may lead to the development of MASLD. This is associated with alterations in plasma lipid levels, PCSK9 levels, and liver LDLR expression. These findings shed new light on the pathogenesis of MASLD, suggesting that excessive caloric intake is not a necessary condition for the onset of this liver pathology.

Despite the development of advanced imaging techniques and blood biomarkers, histopathology remains the gold standard for the diagnosis of MASH and MASLD due to its ability to reveal precise histological features^17,18^. In terms of biomarkers, one of the hallmarks of MASH and MASLD in humans is elevated. Yet, these tests are commonly elevated in MASH but do not often show significant increases in MASLD^19^. Additionally, several studies found no correlation between disease severity and aminotransferase levels in liver diseases^20,21^.

In the present study, liver histopathology revealed that typical MASH changes were present in over half of the animals on the HFD and a quarter on the HDD (Table 3). In contrast, none of the control animals exhibited MASH changes in the liver. Blood AST and ALT levels were not elevated in the HDD and HFD groups compared to the control group. The biochemistry findings highlight conflicting data from other studies in animal models of MASH and MASLD. For instance, Ganbold et al. reported an increase in AST/ALT levels in mice with MASH feed high fat diet (60% cal from fat) and concomitant increase in body weight in these animals relative to controls^22^. In comparison Toita et al. show that mice with normal body weight and obese mice both on a high-fat diet have similar aminotransferases levels, but the obese group had greater liver steatosis^23^. Also, in other studies no increase in aminotransferases were observed in animals with concurrent MASLD/MASH^24,25^.

In the present study, we found significantly elevated LDL levels in the HFD group compared to the HDD and control groups, consistent with other studies showing a similar positive correlation^26,27^. There is likely a causative relationship between plasma LDL and MASLD^28–31^. Notably, in humans and laboratory animals, MASLD is associated with hypercholesterolemia^32,33^. In contrast, a high-carbohydrate diet in humans^34^and mice^13^ produced MASLD without a concomitant increase in plasma LDL, which is in line with our findings in HDD rats.

Plasma LDL levels depend on a complex interplay between dietary delivery and endogenous production of cholesterol^35,36^. The interaction between LDLR and PCSK9 is crucial for transporting plasma cholesterol into hepatocytes. LDLR moves cholesterol into hepatocytes, while PCSK9 degrades internalized LDLR, reducing their availability on the cell membrane^37,38^. In rats fed with a high-fat diet, we found an increased liver LDLR protein and lower plasma PCSK9 levels, suggesting an adaptive mechanism to transport excess plasma LDL to the liver (Fig. 2; Table 2). Previous animal studies provide conflicting results regarding the effect of a high-fat diet on PCSK9. Grzegorczyk et al. showed no effect of a high-fat diet on the serum levels of PCSK9 in rats^39^. In contrast, Lebeau and collaborators found increased PCSK9 levels in mice fed a high-fat diet^40^. Human studies point to either protective^41–43^or neutral effect^44^ of the inhibition of PCSK9 and increasing LDLR in MASLD.

In our study, unlike HFD rats, HDD rats showed normal plasma LDL levels, lower liver LDLR expression, and higher plasma PCSK9 levels. Other studies have shown that a high-fructose diet decreases serum PCSK9 in mice but increases it in hamsters^45^. The research on young male volunteers showed that high fructose intake for 7 days increased plasma PCSK9 levels, independent of cholesterol synthesis^46^. Altogether, these findings suggest that different mechanisms regulate the development of MASLD under excessive carbohydrate or fat intake.

Previous human studies have shown that fat-rich or carbohydrate-rich diets can lead to metabolic disorders, including MASLD^47–50^. Animal studies also demonstrate that excess dietary fats and carbohydrates contribute to liver steatosis^13,15,51–53^. For instance, Jensen et al. found dietary fat significantly drives MASLD development in animals more than fructose^15^. However, these studies involved unrestricted caloric intake, leading to obesity.

There is a lack of research on the effects of dietary fat on PCSK9, and liver steatosis without obesity in a single experiment, particularly in rats. Our study is the first to show that a high-fat diet with normal caloric intake, without causing obesity, can develop MASH in rats. Further investigation is needed, focusing on obesity, MASLD/MASH, and a high-fat diet.

Finally, the effect of sex on the development of MASH in laboratory animals is unclear. In our study, female rats on HFD and HDD diets showed more steatosis and a higher proportion of MASH than males, but these differences were not significant. Plasma lipid levels were also similar between sexes. Previous research without caloric restriction found that a high-fat diet, alone or with high sucrose, caused more liver changes and steatosis in male rats^54,55^. However, both male and female mice on a high-fructose diet developed similar liver steatosis, with males showing more hepatic inflammation^56^. Overall, no strong evidence suggests sex differences in vulnerability to MASH in laboratory rodents, and further studies are needed.

A limitation of our study is that we explored a limited number of molecular pathways potentially affected by a high-fat and high-sugar diet, which may contribute to the development of MASLD/MASH. While we demonstrated that a high-fat diet impacted the LDLR/PCSK9 system, a broader investigation, including interleukins, collagens, and metalloproteinases, is needed to better understand the mechanisms involved in diet-induced liver pathology.

In summary, our study shows that normal caloric intake with a high-fat or high-disaccharide diet induces MASLD or MASH in rats, independent of body weight and sex. Thus, obesity and excessive caloric intake are not necessary for MASLD/MASH development. These findings underscore the importance of a balanced diet for maintaining liver function and lipid homeostasis.

### Methods

#### Animals and experimental design

The experiments were performed according to the Directive 2010/63 EU on the protection of animals used for scientific purposes and approved by the Local Bioethical Committee (no: WAW2/061/2021). In addition, all methods were carried out in accordance with relevant guidelines and regulations, ensuring compliance with ethical standards for animal research. All experiments were conducted in accordance with the aforementioned guidelines and regulations, and in particular in accordance with the ARRIVE guidelines. 6-week-old rats were housed in polypropylene cages with environmental enrichment, 12 h light/12 h dark cycle, temperature 22–23◦C, humidity 45–55%. Experiments were performed on male (*n* = 22) and female (*n* = 22) Sprague-Dawley rats from Central Laboratory for Experimental Animals, Medical University of Warsaw (Poland) were randomly allocated into three groups, control group (C) *n* = 16 receiving standard laboratory diet (C 1090–10, Altromin, Lage, Germany); high-disaccharide diet (HDD) *n* = 14 receiving disaccharide-rich diet (C 1010, Altromin, Lage, Germany); high-fat diet (HFD) *n* = 14 receiving fat-rich diet (C 1090–45, Altromin, Lage, Germany). Table 4presents the nutritive values for each diet. Detailed specifications for each diet are available in Supplementary Files 1–3. All experimental groups received the same caloric dose, adjusted to body weight gain as rats were growing. Rats were weighed weekly and their energy requirements were calculated based on their weights and the amount of feed (g) that corresponded to the calculated caloric value was given each day. The energy requirements were calculated according to Nutrient Requirements of Laboratory Animals^16^. The rats were maintained in a cage with a predetermined amount of food based on their body weight. The remaining food was weighed daily, and the unconsumed amount was totaled and converted into calorie content. The total uneaten food was subtracted from the total provided food to determine the actual energy intake for each rat throughout the experiment. The average total calorie intake is presented in Supplementary Table 1. Table 1 shows the energy intake per 100 g of body weight, calculated from metabolic cages at the start and end of the experiment. The animals were fasted for 6 h before tissue sample collection. Rats were sacrificed by cervical dislocation at the end of experiment.

**Table 4:** Nutritive value, crude nutrients and metabolized energy of each diet.

	Control Diet	HDD	HFD
Metabolized energy [kcal/kg]	3,514	3,772	4,497
Fat [%]	10	12	45
Protein [%]	24	18	18
Carbohydrates [%]	66	70	37
Monosaccharides [mg/kg]	15.143	66.500	102.200
Disaccharides [mg/kg]	117.705	441.105	50.355
Polysaccharides [mg/kg]	427.227	133.527	229.252
Moisture [%]	7.9	5.0	3.9
Crude ash [%]	4.3	4.2	3.9
Crude fiber [%]	3.1	1.5	5.6
Crude fat [%]	4.0	5.0	22.6
Crude protein [%]	20.7	17.1	20.8
Nitrogen free extractives [%]	60	67.2	43.2

All diets were purchased from Altromin, Lage, Germany; Full nutritive value and chemical composition of each diet is presented in Supplementary files 1–3.

## Metabolic experiments

The rats were maintained for 2 days in metabolic cages to evaluate their 24-h fluid and energy balance prior to and after the main section of the study. Body mass, energy and water intake, and urine output were measured at the start and at the end of the experiment.

### Biochemistry

Biochemical analysis of serum (total cholesterol, low-density lipoprotein (LDL-C), high-density lipoprotein (HDL-C), triglycerides, sodium, potassium, aspartate aminotransferase (AST), alanine transaminase (ALT) was performed using a Cobas 6000 analyzer (Roche Diagnostics, Indianapolis, Indiana, USA). The laboratory conducting the tests has obtained validation and adheres to the ISO9001 system in the field of biochemical diagnostics.

### Serum PCSK9 level

A rat serum commercial ELISA kit (LS-F22819, LSBio, USA) was used to evaluate PCSK9. The assay was performed according to the manufacturer’s protocol.

### Histopathology of the liver

Liver tissues were collected from the 3 groups of animals: control (*n* = 15, one sample was damage), HDD (*n* = 14) and HFD (*n*= 14). Tissues were fixed in 10% buffered formalin for 48 h, after which they were subjected to the standard paraffin technique, passing the tissues through a series of alcohols, xylene, to paraffin. The obtained paraffin sections of the liver tissues were stained with standard hematoxylin-eosin staining, Trichrome-Masson staining (BioOptica, Italy). Moreover, immunohistochemical staining for CD3 (CONFIRM anti-CD3 (2GV6) Rabbit Monoclona - Kat. No. 05278422001; Roche Diagnostics, Germany) was performed to detect T lymphocytes using the BenchMark ULTRA IHC/ISH System automatic stainer (Roche Diagnostics, Germany). Histological slides were assessed under x10, x40 and x100 objective magnification using a microscope Axiolab A5 with Axiocam 208 color. Micro-photo documentation was made using ZEN 3.0 software (Zeiss, Jena, Germany). Two Doctors of Veterinary Medicine (DVM) specializing in laboratory animal pathology performed the histopathological evaluations. The examination was blinded. The histopathological examination included a scalar score of 0–3 for such changes as steatosis, lobular inflammation, and fibrosis and 0–2 for ballooning hepatocytes, as described by Carreres et al^33^.

### Western blot analysis

Liver samples were lysed in histidine-sucrose buffer (30mM histidine, 250mM sucrose, 2mM ethylenediaminetetraacetic acid (EDTA), proteases inhibitors, phenylmethylsulfonyl fluoride (PMSF), pH 7.4). Protein concentration was determined using the Bradford Protein Assay (Bio-Rad, Hercules, USA). Laemmli sample buffer 4x was added to all samples. To determine the levels of LDLR and PCSK9, all samples were resolved by electrophoresis on 10% SDS/PAGE gels for PCSK9 and 8% for LDLR. Beta Actin served as a reference protein.

Resolved proteins were transferred onto PVDF membranes (Bio-rad, Hercules, USA), blocked with skimmed milk and incubated with respective primary and secondary antibodies (Supplementary Table 2). Target protein expression was quantified relative to beta-actin using a ChemiDoc MP Imaging System with Quantity One software (Bio-rad, Hercules, USA). Uncropped blots shown in Supplementary Fig. 3.

### Statistical analysis

Data normality tests including the Shapiro-Wilk were performed. All the data were analyzed with one-way analysis of variance (ANOVA), followed by post-hoc Tukey’s test or non-parametric Kruskal-Wallis test for multiple comparisons using GraphPad Prism software (GraphPad, San Diego, USA), *P* < 0.05 was considered significant. Data are presented as means ± Standard Error.

## Electronic supplementary material

Below is the link to the electronic supplementary material.

Supplementary Material 1

Supplementary Material 2

Supplementary Material 3

Supplementary Material 4

## Data Availability

All data generated in this study are contained within the article or supplementary material file.

## Abbreviations

ALT: Alanine transaminase
AST: Aspartate aminotransferase
BW: Body weight
EDTA: Ethylenediaminetetraacetic acid
HDD: High-disaccharide diet
HDL: High-density lipoprotein
HFD: High-fat diet
LDL: Low-density lipoprotein
LDLR: Low-density lipoprotein receptor
MASH: Metabolic dysfunction-associated steatohepatitis
MASLD: Metabolic dysfunction-associated steatotic liver disease
NAFLD: Non-Alcoholic Fatty Liver Disease
NASH: Non-Alcoholic Steatohepatitis
PCSK9: Proprotein convertase subtilisin/kexin type 9
PMSF: Phenylmethylsulfonyl fluoride
