# Supplementary material for: Normal caloric intake with high-fat diet induces metabolic dysfunction-associated steatotic liver disease and dyslipidemia without obesity in rats
Source: Sci Rep. 2024 Oct 1;14:22796. doi: 10.1038/s41598-024-74193-y (PMC11445425; doi:10.1038/s41598-024-74193-y)
Supplement: Supplementary file 1 — Supplementary Material 1 [file 41598_2024_74193_MOESM1_ESM.pdf]

C 1090 - 10

control diet with w/10% energy from fat

Metabolized energy

Content	Value		unit
Fat	358	(10%)	kcal/kg
Protein	828	(24%)	kcal/kg
Carbonhydrates	2,329	(66%)	kcal/kg

crude nutrients and moisture

Content	Value		unit
Moisture	79,090	(7.9%)	mg/kg
Crude Ash	43,274	(4.3%)	mg/kg
Crude Fibre	30,829	(3.1%)	mg/kg
Crude Fat	39,725	(4.0%)	mg/kg
Crude Protein	206,900	(20.7%)	mg/kg
Nitrogenfree extractives	600,183	(60%)	mg/kg

Carbonhydrates

Content	Value	unit
Monosaccharides	15,143	mg/kg
Disaccharides	117,705	mg/kg
Polysaccharides	427,227	mg/kg

Minerals

Content	Value	unit
Calcium	7,402	mg/kg
Potassium	5,703	mg/kg
Magnesium	558	mg/kg
Sodium	1,890	mg/kg
Phosphorus	6,522	mg/kg

Altromin Spezialfutter GmbH & Co. KG
Im Seelenkamp 20, 32791 Lage, Germany
info@altromin.de - www.altromin.com

Trace elements

Content	Value	unit
Aluminium	3.61	mg/kg
Chlorine	2,992.50	mg/kg
Iron	134.70	mg/kg
Flourine	3.13	mg/kg
Iodine	0.41	mg/kg
Cobalt	0.12	mg/kg
Copper	4.46	mg/kg
Manganese	75.75	mg/kg
Molybdenum	0.15	mg/kg
Sulfur	2,779.54	mg/kg
Selenium	0.29	mg/kg
Zinc	24.16	mg/kg

Added vitamins

Content	Value	unit
Vitamin A	15,000	IU/kg
Vitamin D3	500	IU/kg
Vitamin E	150	mg/kg
Vitamin K3	10	mg/kg
Vitamin B1	20	mg/kg
Vitamin B2	20	mg/kg
Vitamin B6	15	mg/kg
Vitamin B12	43	µg/kg
Nicotinic acid	50	mg/kg
Pantothenic acid	50	mg/kg
Folic acid	10	mg/kg
Biotin	201	µg/kg
Choline chloride	1,013	mg/kg
Vitamin C	20	mg/kg

Altromin Spezialfutter GmbH & Co. KG
Im Seelenkamp 20, 32791 Lage, Germany
info@altromin.de - www.altromin.com

Amino acids

Content	Value	unit
Alanine	3,134	mg/kg
Arginine	11,620	mg/kg
Aspartic acid	4,774	mg/kg
Cystine	3,747	mg/kg
Glutaminc acid	28,926	mg/kg
Glycine	4,141	mg/kg
Histidine	6,283	mg/kg
Isoleucine	8,898	mg/kg
Leucine	17,033	mg/kg
Lysine	20,604	mg/kg
Methionine	8,609	mg/kg
Phenylalanine	8,654	mg/kg
Proline	15,389	mg/kg
Serine	6,481	mg/kg
Threonine	8,608	mg/kg
Tryptophan	2,404	mg/kg
Tyrosine	11,073	mg/kg
Valine	4,320	mg/kg

Fatty acid

Content	Value	unit
Arachidic acid C-20:0	340	mg/kg
Eicosanoic acid C-20:1	153	mg/kg
Alpha-Linolenic acid C-18:3	333	mg/kg
Linolenic acid C-18:2	2,059	mg/kg
Palmitic acid C-16:0	5,240	mg/kg
Stearic acid C-18:0	3,793	mg/kg
Oleic acid C-18:1	11,300	mg/kg

Altromin Spezialfutter GmbH & Co. KG
Im Seelenkamp 20, 32791 Lage, Germany
info@altromin.de - www.altromin.com
