# Supplementary material for: Normal caloric intake with high-fat diet induces metabolic dysfunction-associated steatotic liver disease and dyslipidemia without obesity in rats
Source: Sci Rep. 2024 Oct 1;14:22796. doi: 10.1038/s41598-024-74193-y (PMC11445425; doi:10.1038/s41598-024-74193-y)
Supplement: Supplementary file 2 — Supplementary Material 2 [file 41598_2024_74193_MOESM2_ESM.pdf]

C 1010

carbohydrate rich diet

Metabolized energy

Content	Value		unit
Fat	454	(12%)	kcal/kg
Protein	683	(18%)	kcal/kg
Carbonhydrates	2,635	(70%)	kcal/kg

crude nutrients and moisture

Content	Value		unit
Moisture	49,870	(5.0%)	mg/kg
Crude Ash	41,708	(4.2%)	mg/kg
Crude Fibre	15,170	(1.5%)	mg/kg
Crude Fat	50,450	(5.0%)	mg/kg
Crude Protein	170,750	(17.1%)	mg/kg
Nitrogenfree extractives	672,052	(67.2%)	mg/kg

Carbonhydrates

Content	Value	unit
Monosaccharides	66,500	mg/kg
Disaccharides	441,105	mg/kg
Polysaccharides	133,527	mg/kg

Minerals

Content	Value	unit
Calcium	6,193	mg/kg
Potassium	4,736	mg/kg
Magnesium	480	mg/kg
Sodium	1,665	mg/kg
Phosphorus	5,557	mg/kg

Altromin Spezialfutter GmbH & Co. KG
Im Seelenkamp 20, 32791 Lage, Germany
info@altromin.de - www.altromin.com

Trace elements

Content	Value	unit
Aluminium	2.41	mg/kg
Chlorine	2,420.00	mg/kg
Iron	119.26	mg/kg
Flourine	2.78	mg/kg
Iodine	0.36	mg/kg
Cobalt	0.09	mg/kg
Copper	3.88	mg/kg
Manganese	67.24	mg/kg
Molybdenum	0.13	mg/kg
Sulfur	2,484.52	mg/kg
Selenium	0.25	mg/kg
Zinc	21.29	mg/kg

Added vitamins

Content	Value	unit
Vitamin A	15,000	IU/kg
Vitamin D3	500	IU/kg
Vitamin E	180	mg/kg
Vitamin K3	10	mg/kg
Vitamin B1	20	mg/kg
Vitamin B2	20	mg/kg
Vitamin B6	15	mg/kg
Vitamin B12	41	µg/kg
Nicotinic acid	50	mg/kg
Pantothenic acid	50	mg/kg
Folic acid	10	mg/kg
Biotin	201	µg/kg
Choline chloride	1,012	mg/kg
Vitamin C	20	mg/kg

Altromin Spezialfutter GmbH & Co. KG
Im Seelenkamp 20, 32791 Lage, Germany
info@altromin.de - www.altromin.com

Amino acids

Content	Value	unit
Alanine	2,376	mg/kg
Arginine	9,736	mg/kg
Aspartic acid	3,455	mg/kg
Cystine	3,156	mg/kg
Glutaminc acid	23,314	mg/kg
Glycine	3,060	mg/kg
Histidine	5,221	mg/kg
Isoleucine	7,149	mg/kg
Leucine	14,531	mg/kg
Lysine	17,344	mg/kg
Methionine	7,185	mg/kg
Phenylalanine	7,077	mg/kg
Proline	12,586	mg/kg
Serine	5,169	mg/kg
Threonine	7,082	mg/kg
Tryptophan	1,965	mg/kg
Tyrosine	9,203	mg/kg
Valine	3,206	mg/kg

Fatty acid

Content	Value	unit
Arachidic acid C-20:0	50	mg/kg
Eicosanoic acid C-20:1	150	mg/kg
Alpha-Linolenic acid C-18:3	150	mg/kg
Linolenic acid C-18:2	28,500	mg/kg
Palmitic acid C-16:0	2,500	mg/kg
Stearic acid C-18:0	1,350	mg/kg
Oleic acid C-18:1	13,500	mg/kg

Altromin Spezialfutter GmbH & Co. KG
Im Seelenkamp 20, 32791 Lage, Germany
info@altromin.de - www.altromin.com
