# Supplementary material for: Normal caloric intake with high-fat diet induces metabolic dysfunction-associated steatotic liver disease and dyslipidemia without obesity in rats
Source: Sci Rep. 2024 Oct 1;14:22796. doi: 10.1038/s41598-024-74193-y (PMC11445425; doi:10.1038/s41598-024-74193-y)
Supplement: Supplementary file 3 — Supplementary Material 3 [file 41598_2024_74193_MOESM3_ESM.pdf]

C 1090 - 45

obesity-inducing diet with w/45% energy from fat (22%fat)

Metabolized energy

Content		Value	unit
Fat	2,037	(45%)	kcal/kg
Protein	831	(18%)	kcal/kg
Carbonhydrates	1,629	(37%)	kcal/kg

crude nutrients and moisture

Content		Value	unit
Moisture	39,075	(3.9%)	mg/kg
Crude Ash	39,100	(3.9%)	mg/kg
Crude Fibre	55,707	(5.6%)	mg/kg
Crude Fat	226,300	(22.6%)	mg/kg
Crude Protein	207,775	(20.8%)	mg/kg
Nitrogenfree extractives	432,043	(43.2%)	mg/kg

Carbonhydrates

Content	Value	unit
Monosaccharides	102,200	mg/kg
Disaccharides	50,355	mg/kg
Polysaccharides	229,252	mg/kg

Minerals

Content	Value	unit
Calcium	7,988	mg/kg
Potassium	7,155	mg/kg
Magnesium	651	mg/kg
Sodium	2,363	mg/kg
Phosphorus	5,882	mg/kg

Altromin Spezialfutter GmbH & Co. KG
Im Seelenkamp 20, 32791 Lage, Germany
info@altromin.de - www.altromin.com

Trace elements

Content	Value	unit
Aluminium	2.97	mg/kg
Chlorine	4,220.00	mg/kg
Iron	119.31	mg/kg
Flourine	3.33	mg/kg
Iodine	0.31	mg/kg
Cobalt	0.10	mg/kg
Copper	3.78	mg/kg
Manganese	67.36	mg/kg
Molybdenum	0.19	mg/kg
Sulfur	1,522.08	mg/kg
Selenium	0.19	mg/kg
Zinc	17.43	mg/kg

Added vitamins

Content	Value	unit
Vitamin A	15,000	IU/kg
Vitamin D3	500	IU/kg
Vitamin E	150	mg/kg
Vitamin K3	10	mg/kg
Vitamin B1	20	mg/kg
Vitamin B2	20	mg/kg
Vitamin B6	15	mg/kg
Vitamin B12	32	µg/kg
Nicotinic acid	50	mg/kg
Pantothenic acid	50	mg/kg
Folic acid	10	mg/kg
Biotin	200	µg/kg
Choline chloride	1,002	mg/kg
Vitamin C	20	mg/kg

Altromin Spezialfutter GmbH & Co. KG
Im Seelenkamp 20, 32791 Lage, Germany
info@altromin.de - www.altromin.com

Amino acids

Content	Value	unit
Alanine	10,977	mg/kg
Arginine	12,804	mg/kg
Aspartic acid	11,759	mg/kg
Cystine	1,465	mg/kg
Glutaminc acid	29,145	mg/kg
Glycine	22,517	mg/kg
Histidine	3,470	mg/kg
Isoleucine	7,154	mg/kg
Leucine	7,553	mg/kg
Lysine	11,782	mg/kg
Methionine	4,416	mg/kg
Phenylalanine	6,978	mg/kg
Proline	19,684	mg/kg
Serine	7,906	mg/kg
Threonine	6,147	mg/kg
Tryptophan	1,300	mg/kg
Tyrosine	5,289	mg/kg
Valine	7,297	mg/kg

Fatty acid

Content	Value	unit
Arachidic acid C-20:0	0	mg/kg
Eicosanoic acid C-20:1	0	mg/kg
Alpha-Linolenic acid C-18:3	1,582	mg/kg
Linolenic acid C-18:2	2,260	mg/kg
Palmitic acid C-16:0	5,017	mg/kg
Stearic acid C-18:0	13,786	mg/kg
Oleic acid C-18:1	38,872	mg/kg

Altromin Spezialfutter GmbH & Co. KG
Im Seelenkamp 20, 32791 Lage, Germany
info@altromin.de - www.altromin.com
