# Supplementary material for: Normal caloric intake with high-fat diet induces metabolic dysfunction-associated steatotic liver disease and dyslipidemia without obesity in rats
Source: Sci Rep. 2024 Oct 1;14:22796. doi: 10.1038/s41598-024-74193-y (PMC11445425; doi:10.1038/s41598-024-74193-y)

Supplementary Table 1. Average total energy and food intake.

|  | Average total energy intake /rat/experiment (kcal) | Average total food intake/rat/experiment  (g) |
| --- | --- | --- |
| Control diet | 4560.06 | 1297.68 |
| HDD | 4520.90 | 1198.54 |
| HFD | 4368.76 | 971.48 |

Supplementary Table 2. List of antibodies used for Western blot analyses.

| **Target**  **protein** | **Primary Ab** | **Dilution** | **Secondary Ab** | **Dilution** |
| --- | --- | --- | --- | --- |
| PCSK9 | Rabbit polyclonal Invitrogen. PA5-115725 | 1:500 | Goat anti-rabbit, Abcam  ab97048-1 | 1:10.000 |
| LDLr | Rabbit polyclonal Abcam  ab30532 | 1:1000 | Goat anti-rabbit, Abcam  ab97048-1 | 1:10.000 |
| Beta-Actin | Goat polyclonal  Abcam  ab8229-100 | 1:2000 | Donkey anti-goat, Abcam  ab97107-1 | 1:10.000 |

|  | Male | | | | | | Female | | | | | |
| --- | --- | --- | --- | --- | --- | --- | --- | --- | --- | --- | --- | --- |
|  | Controls | | HDD | | HFD | | Controls | | HDD | | HFD | |
|  | start | end | start | end | start | end | start | end | start | end | start | end |
| Initial body weight (g) | 208.67±  9.41 | N/A | 206.88  ±  5.97 | N/A | 203.44±  4.51 | N/A | 166.70±  5.25 | N/A | 161.41  ±  4.38 | N/A | 165.5±  6.40 | N/A |
| Final body weight (g) | N/A | 470.61  ±  21.88 | N/A | 482.27  ±  10.53 | N/A | 456.14  ±  7.71 | N/A | 291.81  ±  6.42 | N/A | 289.07  ±  10.70 | N/A | 281.89  ±  10.89 |
| Energy intake (kcal/day/100 g BW) | 38.60  ±  0.28 | 15.12  ±  0.13 | 39.36  ±  0.31 | 14.99±  0.13 | 37.87  ±  0.26 | 16.22  ±  0.13 | 36.07  ±  0.28 | 17.17  ±  0.20 | 36.89  ±  0.37 | 17.64  ±  0.17 | 38.75  ±  0.37 | 17.46  ±  0.19 |
| Water intake g/100g BW | 13.55  ±  0.48 | 4.42  ±  0.30 | 14.24  ±  0.33 | 3.16  ±  0.16* | 13.73  ±  0.17 | 3.89  ±  0.13 | 13.55  ±  0.67 | 7.99  ±  0.84 | 12.06  ±  0.55 | 4.58  ±  0.29  * | 15.34  ±  0.87 | 6.70  ±  0.72 |
| Urine output g/100g BW | 3.39  ±  0.24 | 2.30  ±  0.10 | 3.53  ±  0.18 | 1.58  ±  0.08** | 3.48  ±  0.17 | 1.59  ±  0.04** | 3.50  ±  0.23 | 3.38  ±  0.62 | 2.90  ±  0.20 | 1.97  ±  0.29** | 3.94  ±  0.54 | 2.73  ±  0.36** |
| Stool output g/100g BW | 5.66  ±  0.22 | 0.76  ±  0.06 | 5.51  ±  0.31 | 0.48  ±  0.04 | 5.44  ±  0.17 | 0.93  ±  0.12 | 4.84  ±  0.20 | 1.04  ±  0.09 | 6.24  ±  0.38 | 0.46  ±  0.04* | 5.77  ±  0.33 | 1.01  ±  0.11 |

Supplementary Table 3. Metabolic parameters at the beginning and at the end of experiments calculated per 100g of body weight (BW) in Sprague Dawley male and female rats maintained on control, high-disaccharide diet (HDD) and high-fat diet (HFD) for 12 weeks.

* *P*<0.05. ***P*<0.01 vs. controls; Means ± SE are presented.

Supplementary Table 4. Lipid profile. glucose. sodium. potassium and aminotransferases in Sprague Dawley male and female rats maintained on control, high-disaccharide diet (HDD) and high-fat diet (HFD) for 12 weeks.

|  | Male | | | Female | | |
| --- | --- | --- | --- | --- | --- | --- |
|  | Controls | HDD | HFD | Controls | HDD | HFD |
| Total cholesterol  (mg/dL) | 58.50  ±  1.93 | 72.14**  ±  4.03 | 61.14  ±  2.69 | 57.37  ±  3.02 | 61.00  ±  3.57 | 65.00  ±  3.57 |
| LDL  (mg/dL) | 9.15  ±  0.37 | 9.78  ±  1.05 | 11.32*  ±  0.68 | 8.27  ±  0.36 | 7.36  ±  0.54 | 11.38*##  ±  0.90 |
| HDL  (mg/dL) | 43.12  ±  1.96 | 50.28  ±  3.98 | 47.26  ±  2.16 | 48.61  ±  2.60 | 41.02  ±  5.49 | 56.59  ±  3.90 |
| Triglycerides  (mg/dL) | 157.50  ±  13.22 | 205.00  ±  24.50 | 131.42  ±  10.60 | 104.25  ±  12.07 | 150.71  ±  23.88 | 99.86  ±  11.65 |
| Glucose  (mg/dL) | 213.62  ±  13.95 | 240.71  ±  12.24 | 190.50  ±  13.41 | 214.12  ±  17.76 | 236.14  ±  16.98 | 254.42  ±  14.49 |
| Sodium (mmol/l) | 139.75  ±  1.62 | 137.00  ±  0.92 | 138.42  ±  1.06 | 137.75  ±  0.79 | 139.14  ±  1.86 | 138.66  ±  0.57 |
| Potassium (mmol/l) | 4.65  ±  0.08 | 4.73  ±  0.13 | 4.59  ±  0.20 | 4.10  ±  0.08 | 4.48  ±  0.23 | 5.17  ±  0.73 |
| AST | 147.83  ±  16.35 | 152.5  ±  15.16 | 97.5  ±  8.86 | 123.00  ±  12.89 | 144.66  ±  10.09 | 237.5  ±  36.47 |
| ALT | 25.6  ±  3.21 | 27.30  ±  1.39 | 24.25  ±  5.78 | 18.75  ±  1.28 | 13.66  ±  1.16 | 14.5  ±  0.56 |
| Creatinine clearance (ml/min) | 1.49  ±  0.15 | 1.05  ±  0.07 | 1.35  ±  0.14 | 0.81  ±  0.07 | 0.65  ±  0.05 | 0.77  ±  0.07 |

* *P*<0.05. ***P*<0.01 vs. controls; #*P*<0.05. ##*P*<0.01 HDD vs. HFD Means ± SE are presented.

Supplementary table 5. Results of histopathological scalar assessments of the liver for the three study groups in male and female separately.

|  | **SCORE** | **Control**  **(n=15)** | | **HDD**  **(n=14)** | | **HFD**  **(n=14)** | |
| --- | --- | --- | --- | --- | --- | --- | --- |
|  |  | Male  **(n=7)** | Female  **(n=8)** | Male  **(n=7)** | Female  **(n=7)** | Male  **(n=7)** | Female  **(n=7)** |
| Steatosis  scoring | 0  I  II  III | 7  0  0  0 | 4  2  2  0 | 6  1  0  0 | 1  2  4  0 | 5  2  0  0 | 0  0  3  4 |
| Inflammation  scoring | 0  I  II  III | 5  2  0  0 | 5  3  0  0 | 1  4  2  0 | 1  2  3  1 | 3  3  1  0 | 0  5  1  1 |
| Ballooning hepatocytes  scoring | 0  I  II | 6  1  0 | 8  0  0 | 2  4  1 | 2  2  3 | 1  5  1 | 1  6  0 |
| Fibrosis  scoring | 0  I  II  III | 7  0  0  0 | 6  2  0  0 | 6  1  0  0 | 6  1  0  0 | 7  0  0  0 | 2  5  0  0 |
|  |  |  |  |  |  |  |  |

For each histopathological criterion, scoring points were shown; values indicate the number of rats.

Two-way analysis of variance.

| Table Analyzed | Water intake-start | |  |  |  |
| --- | --- | --- | --- | --- | --- |
|  |  |  |  |  |  |
| Two-way ANOVA | |  |  |  |  |
|  |  |  |  |  |  |
| Source of Variation | % of total variation | P value |  |  |  |
| Interaction | 11 | 0,0873 |  |  |  |
| sex | 0,86 | 0,5284 |  |  |  |
| diet | 6,44 | 0,2309 |  |  |  |
|  |  |  |  |  |  |
| Source of Variation | P value summary | Significant? | |  |  |
| Interaction | ns | No |  |  |  |
| sex | ns | No |  |  |  |
| diet | ns | No |  |  |  |
|  |  |  |  |  |  |
| Source of Variation | Df | Sum-of-squares | Mean square | F |  |
| Interaction | 2 | 26,8 | 13,4 | 2,602 |  |
| sex | 1 | 2,085 | 2,085 | 0,4048 |  |
| diet | 2 | 15,7 | 7,849 | 1,524 |  |
|  |  |  |  |  |  |
| Bonferroni posttests | |  |  |  |  |
| male vs female |  |  |  |  |  |
| diet | Difference | t | P value | Summary |  |
| Controls | 0,2589 | 0,2282 | P > 0,05 | ns |  |
| HDD | -2,619 | 2,23 | P > 0,05 | ns |  |
| HFD | 1,047 | 0,8292 | P > 0,05 | ns |  |

| Table Analyzed | Stool-start | |  |  |  |
| --- | --- | --- | --- | --- | --- |
|  |  |  |  |  |  |
| Two-way ANOVA | |  |  |  |  |
|  |  |  |  |  |  |
| Source of Variation | % of total variation | P value |  |  |  |
| Interaction | 7,81 | 0,1976 |  |  |  |
| sex | 0,15 | 0,8023 |  |  |  |
| diet | 6,91 | 0,2369 |  |  |  |
|  |  |  |  |  |  |
| Source of Variation | P value summary | Significant? | |  |  |
| Interaction | ns | No |  |  |  |
| sex | ns | No |  |  |  |
| diet | ns | No |  |  |  |
|  |  |  |  |  |  |
| Source of Variation | Df | Sum-of-squares | Mean square | F |  |
| Interaction | 2 | 4,713 | 2,356 | 1,695 |  |
| sex | 1 | 0,08841 | 0,08841 | 0,06359 |  |
| diet | 2 | 4,165 | 2,082 | 1,498 |  |
|  |  |  |  |  |  |
| Bonferroni posttests | |  |  |  |  |
| male vs female |  |  |  |  |  |
| diet | Difference | t | P value | Summary |  |
| Controls | -0,7917 | 1,343 | P > 0,05 | ns |  |
| HDD | 0,7295 | 1,157 | P > 0,05 | ns |  |
| HFD | 0,3355 | 0,5115 | P > 0,05 | ns |  |

| Table Analyzed | urine output-start | |  |  |  |
| --- | --- | --- | --- | --- | --- |
|  |  |  |  |  |  |
| Two-way ANOVA | |  |  |  |  |
|  |  |  |  |  |  |
| Source of Variation | % of total variation | P value |  |  |  |
| Interaction | 2,45 | 0,6408 |  |  |  |
| sex | 0,03 | 0,9148 |  |  |  |
| diet | 2,68 | 0,6147 |  |  |  |
|  |  |  |  |  |  |
| Source of Variation | P value summary | Significant? | |  |  |
| Interaction | ns | No |  |  |  |
| sex | ns | No |  |  |  |
| diet | ns | No |  |  |  |
|  |  |  |  |  |  |
| Source of Variation | Df | Sum-of-squares | Mean square | F |  |
| Interaction | 2 | 1,297 | 0,6485 | 0,4508 |  |
| sex | 1 | 0,01669 | 0,01669 | 0,01161 |  |
| diet | 2 | 1,42 | 0,7099 | 0,4935 |  |
|  |  |  |  |  |  |
| Bonferroni posttests | |  |  |  |  |
| male vs female |  |  |  |  |  |
| diet | Difference | t | P value | Summary |  |
| Controls | -0,1867 | 0,3113 | P > 0,05 | ns |  |
| HDD | -0,4023 | 0,5729 | P > 0,05 | ns |  |
| HFD | 0,4662 | 0,6987 | P > 0,05 | ns |  |

| Table Analyzed | Energy intake-start | |  |  |  |
| --- | --- | --- | --- | --- | --- |
|  |  |  |  |  |  |
| Two-way ANOVA | |  |  |  |  |
|  |  |  |  |  |  |
| Source of Variation | % of total variation | P value |  |  |  |
| Interaction | 2,27 | 0,6747 |  |  |  |
| sex | 0 | 0,9705 |  |  |  |
| diet | 1,05 | 0,8329 |  |  |  |
|  |  |  |  |  |  |
| Source of Variation | P value summary | Significant? | |  |  |
| Interaction | ns | No |  |  |  |
| sex | ns | No |  |  |  |
| diet | ns | No |  |  |  |
|  |  |  |  |  |  |
| Source of Variation | Df | Sum-of-squares | Mean square | F |  |
| Interaction | 2 | 20,25 | 10,13 | 0,3981 |  |
| sex | 1 | 0,03519 | 0,03519 | 0,001383 |  |
| diet | 2 | 9,35 | 4,675 | 0,1838 |  |
|  |  |  |  |  |  |
| Bonferroni posttests | |  |  |  |  |
| male vs female |  |  |  |  |  |
| diet | Difference | t | P value | Summary |  |
| Controls | -1,613 | 0,5921 | P > 0,05 | ns |  |
| HDD | -0,5732 | 0,2105 | P > 0,05 | ns |  |
| HFD | 2,002 | 0,6484 | P > 0,05 | ns |  |

| Table Analyzed | initial bw |  |  |  |  |
| --- | --- | --- | --- | --- | --- |
|  |  |  |  |  |  |
| Two-way ANOVA | |  |  |  |  |
|  |  |  |  |  |  |
| Source of Variation | % of total variation | P value |  |  |  |
| Interaction | 0,27 | 0,7546 |  |  |  |
| sex | 59,94 | < 0,0001 |  |  |  |
| diet | 0,42 | 0,6414 |  |  |  |
|  |  |  |  |  |  |
| Source of Variation | P value summary | Significant? | |  |  |
| Interaction | ns | No |  |  |  |
| sex | *** | Yes |  |  |  |
| diet | ns | No |  |  |  |
|  |  |  |  |  |  |
| Source of Variation | Df | Sum-of-squares | Mean square | F |  |
| Interaction | 2 | 155,6 | 77,78 | 0,2825 |  |
| sex | 1 | 34786 | 34786 | 126,4 |  |
| diet | 2 | 245,9 | 122,9 | 0,4465 |  |
|  |  |  |  |  |  |
| Bonferroni posttests | |  |  |  |  |
| male vs female |  |  |  |  |  |
| diet | Difference | t | P value | Summary |  |
| Controls | -41,54 | 7,082 | P<0,001 | *** |  |
| HDD | -41,95 | 6,689 | P<0,001 | *** |  |
| HFD | -36,04 | 5,746 | P<0,001 | *** |  |

| Table Analyzed | Water intake - finish | |  |  |  |
| --- | --- | --- | --- | --- | --- |
|  |  |  |  |  |  |
| Two-way ANOVA | |  |  |  |  |
|  |  |  |  |  |  |
| Source of Variation | % of total variation | P value |  |  |  |
| Interaction | 3,67 | 0,2652 |  |  |  |
| sex | 29,26 | < 0,0001 |  |  |  |
| diet | 15,18 | 0,0069 |  |  |  |
|  |  |  |  |  |  |
| Source of Variation | P value summary | Significant? | |  |  |
| Interaction | ns | No |  |  |  |
| sex | *** | Yes |  |  |  |
| diet | ** | Yes |  |  |  |
|  |  |  |  |  |  |
| Source of Variation | Df | Sum-of-squares | Mean square | F |  |
| Interaction | 2 | 9,529 | 4,765 | 1,375 |  |
| sex | 1 | 75,96 | 75,96 | 21,92 |  |
| diet | 2 | 39,4 | 19,7 | 5,685 |  |
|  |  |  |  |  |  |
| Bonferroni posttests | |  |  |  |  |
| male vs female |  |  |  |  |  |
| diet | Difference | t | P value | Summary |  |
| Controls | 3,67 | 3,943 | P<0,01 | ** |  |
| HDD | 1,42 | 1,427 | P > 0,05 | ns |  |
| HFD | 2,809 | 2,823 | P < 0,05 | * |  |

| Table Analyzed | stool output finish | |  |  |  |
| --- | --- | --- | --- | --- | --- |
|  |  |  |  |  |  |
| Two-way ANOVA | |  |  |  |  |
|  |  |  |  |  |  |
| Source of Variation | % of total variation | P value |  |  |  |
| Interaction | 1,06 | 0,7448 |  |  |  |
| sex | 2,88 | 0,2128 |  |  |  |
| diet | 28,49 | 0,0013 |  |  |  |
|  |  |  |  |  |  |
| Source of Variation | P value summary | Significant? | |  |  |
| Interaction | ns | No |  |  |  |
| sex | ns | No |  |  |  |
| diet | ** | Yes |  |  |  |
|  |  |  |  |  |  |
| Source of Variation | Df | Sum-of-squares | Mean square | F |  |
| Interaction | 2 | 0,07301 | 0,0365 | 0,2969 |  |
| sex | 1 | 0,1974 | 0,1974 | 1,606 |  |
| diet | 2 | 1,956 | 0,978 | 7,954 |  |
|  |  |  |  |  |  |
| Bonferroni posttests | |  |  |  |  |
| male vs female |  |  |  |  |  |
| diet | Difference | t | P value | Summary |  |
| Controls | 0,1641 | 0,936 | P > 0,05 | ns |  |
| HDD | 0,02099 | 0,1108 | P > 0,05 | ns |  |
| HFD | 0,2205 | 1,165 | P > 0,05 | ns |  |

| Table Analyzed | urine output finish | |  |  |  |
| --- | --- | --- | --- | --- | --- |
|  |  |  |  |  |  |
| Two-way ANOVA | |  |  |  |  |
|  |  |  |  |  |  |
| Source of Variation | % of total variation | P value |  |  |  |
| Interaction | 6,1 | 0,2031 |  |  |  |
| sex | 9,23 | 0,031 |  |  |  |
| diet | 19,42 | 0,0096 |  |  |  |
|  |  |  |  |  |  |
| Source of Variation | P value summary | Significant? | |  |  |
| Interaction | ns | No |  |  |  |
| Diet | * | Yes |  |  |  |
| Sex | ** | Yes |  |  |  |
|  |  |  |  |  |  |
| Source of Variation | Df | Sum-of-squares | Mean square | F |  |
| Interaction | 2 | 2,088 | 1,044 | 1,667 |  |
| Diet | 1 | 3,158 | 3,158 | 5,041 |  |
| Sex | 2 | 6,644 | 3,322 | 5,303 |  |
|  |  |  |  |  |  |
|  |  |  |  |  |  |
| Bonferroni posttests | | |  |  |  |
| male vs female |  |  |  |  |  |
| diet | Difference | t | P value | Summary |  |
| Controls | 0,5914 | 1,444 | P > 0,05 | ns |  |
| HDD | -0,02707 | 0,06147 | P > 0,05 | ns |  |
| HFD | 1,092 | 2,555 | P < 0,05 | * |  |

| Table Analyzed | energy intake finish | |  |  |  |
| --- | --- | --- | --- | --- | --- |
|  |  |  |  |  |  |
| Two-way ANOVA | |  |  |  |  |
|  |  |  |  |  |  |
| Source of Variation | % of total variation | P value |  |  |  |
| Interaction | 7,74 | 0,1175 |  |  |  |
| sex | 25,1 | 0,0005 |  |  |  |
| diet | 2,73 | 0,4568 |  |  |  |
|  |  |  |  |  |  |
| Source of Variation | P value summary | Significant? | |  |  |
| Interaction | ns | No |  |  |  |
| sex | *** | Yes |  |  |  |
| diet | ns | No |  |  |  |
|  |  |  |  |  |  |
| Source of Variation | Df | Sum-of-squares | Mean square | F |  |
| Interaction | 2 | 8,558 | 4,279 | 2,274 |  |
| Sex | 1 | 27,75 | 27,75 | 14,74 |  |
| Diet | 2 | 3,014 | 1,507 | 0,8007 |  |
|  |  |  |  |  |  |
| Bonferroni posttests | |  |  |  |  |
| male vs female |  |  |  |  |  |
| Diet | Difference | t | P value | Summary |  |
| Controls | 2,469 | 3,478 | P<0,01 | ** |  |
| HDD | 2,143 | 2,893 | P < 0,05 | * |  |
| HFD | 0,3414 | 0,4366 | P > 0,05 | ns |  |

| Table Analyzed | final bw |  |  |  |  |
| --- | --- | --- | --- | --- | --- |
|  |  |  |  |  |  |
| Two-way ANOVA | |  |  |  |  |
|  |  |  |  |  |  |
| Source of Variation | % of total variation | P value |  |  |  |
| Interaction | 0,33 | 0,1222 |  |  |  |
| sex | 91,67 | < 0,0001 |  |  |  |
| diet | 0,93 | 0,0037 |  |  |  |
|  |  |  |  |  |  |
| Source of Variation | P value summary | Significant? | |  |  |
| Interaction | ns | No |  |  |  |
| sex | *** | Yes |  |  |  |
| diet | ** | Yes |  |  |  |
|  |  |  |  |  |  |
| Source of Variation | Df | Sum-of-squares | Mean square | F |  |
| Interaction | 2 | 2822 | 1411 | 2,157 |  |
| sex | 1 | 778859 | 778859 | 1191 |  |
| diet | 2 | 7871 | 3936 | 6,018 |  |
|  |  |  |  |  |  |
| Bonferroni posttests | |  |  |  |  |
| male vs female |  |  |  |  |  |
| diet | Difference | t | P value | Summary |  |
| Controls | -201,1 | 21,89 | P<0,001 | *** |  |
| HDD | -193,3 | 20 | P<0,001 | *** |  |
| HFD | -174 | 18,01 | P<0,001 | *** |  |

| Table Analyzed | sodium |  |  |  |  |
| --- | --- | --- | --- | --- | --- |
|  |  |  |  |  |  |
| Two-way ANOVA | |  |  |  |  |
|  |  |  |  |  |  |
| Source of Variation | % of total variation | P value |  |  |  |
| Interaction | 9,21 | 0,1648 |  |  |  |
| sex | 0,2 | 0,7763 |  |  |  |
| diet | 0,37 | 0,9271 |  |  |  |
|  |  |  |  |  |  |
| Source of Variation | P value summary | Significant? | |  |  |
| Interaction | ns | No |  |  |  |
| sex | ns | No |  |  |  |
| diet | ns | No |  |  |  |
|  |  |  |  |  |  |
| Source of Variation | Df | Sum-of-squares | Mean square | F |  |
| Interaction | 2 | 42,41 | 21,21 | 1,893 |  |
| sex | 1 | 0,9174 | 0,9174 | 0,08191 |  |
| diet | 2 | 1,699 | 0,8497 | 0,07586 |  |
|  |  |  |  |  |  |
| Bonferroni posttests | | |  |  |  |
| male vs female |  |  |  |  |  |
| diet | Difference | t | P value | Summary |  |
| control | -2 | 1,195 | P > 0,05 | ns |  |
| HDD | 2,792 | 1,545 | P > 0,05 | ns |  |
| HFD | 0,1 | 0,05242 | P > 0,05 | ns |  |

| Table Analyzed | potassium | |  |  |  |
| --- | --- | --- | --- | --- | --- |
|  |  |  |  |  |  |
| Two-way ANOVA | |  |  |  |  |
|  |  |  |  |  |  |
| Source of Variation | % of total variation | P value |  |  |  |
| Interaction | 8,31 | 0,1797 |  |  |  |
| sex | 0,2 | 0,7729 |  |  |  |
| diet | 6,28 | 0,2694 |  |  |  |
|  |  |  |  |  |  |
| Source of Variation | P value summary | Significant? | |  |  |
| Interaction | ns | No |  |  |  |
| sex | ns | No |  |  |  |
| diet | ns | No |  |  |  |
|  |  |  |  |  |  |
| Source of Variation | Df | Sum-of-squares | Mean square | F |  |
| Interaction | 2 | 2,366 | 1,183 | 1,799 |  |
| sex | 1 | 0,05557 | 0,05557 | 0,08449 |  |
| diet | 2 | 1,788 | 0,8938 | 1,359 |  |
|  |  |  |  |  |  |
| Bonferroni posttests | | |  |  |  |
| male vs female |  |  |  |  |  |
| diet | Difference | t | P value | Summary |  |
| control | -0,5462 | 1,347 | P > 0,05 | ns |  |
| HDD | -0,25 | 0,5767 | P > 0,05 | ns |  |
| HFD | 0,5795 | 1,284 | P > 0,05 | ns |  |

| Table Analyzed | cholesterole | |  |  |  |
| --- | --- | --- | --- | --- | --- |
|  |  |  |  |  |  |
| Two-way ANOVA | |  |  |  |  |
|  |  |  |  |  |  |
| Source of Variation | % of total variation | P value |  |  |  |
| Interaction | 10,66 | 0,0735 |  |  |  |
| sex | 2,24 | 0,2851 |  |  |  |
| diet | 14,78 | 0,0293 |  |  |  |
|  |  |  |  |  |  |
| Source of Variation | P value summary | Significant? | |  |  |
| Interaction | ns | No |  |  |  |
| sex | ns | No |  |  |  |
| diet | * | Yes |  |  |  |
|  |  |  |  |  |  |
| Source of Variation | Df | Sum-of-squares | Mean square | F |  |
| Interaction | 2 | 409,9 | 204,9 | 2,798 |  |
| sex | 1 | 86,12 | 86,12 | 1,176 |  |
| diet | 2 | 568,3 | 284,1 | 3,879 |  |
|  |  |  |  |  |  |
| Bonferroni posttests | | |  |  |  |
| male vs female |  |  |  |  |  |
| diet | Difference | t | P value | Summary |  |
| control | -1,125 | 0,2629 | P > 0,05 | ns |  |
| HDD | -11,14 | 2,436 | P > 0,05 | ns |  |
| HFD | 3,857 | 0,8431 | P > 0,05 | ns |  |

| Table Analyzed | LDL |  |  |  |  |
| --- | --- | --- | --- | --- | --- |
|  |  |  |  |  |  |
| Two-way ANOVA | |  |  |  |  |
|  |  |  |  |  |  |
| Source of Variation | % of total variation | P value |  |  |  |
| Interaction | 4,25 | 0,4407 |  |  |  |
| sex | 2,91 | 0,2912 |  |  |  |
| diet | 17,38 | 0,045 |  |  |  |
|  |  |  |  |  |  |
| Source of Variation | P value summary | Significant? | |  |  |
| Interaction | ns | No |  |  |  |
| sex | ns | No |  |  |  |
| diet | * | Yes |  |  |  |
|  |  |  |  |  |  |
| Source of Variation | Df | Sum-of-squares | Mean square | F |  |
| Interaction | 2 | 11,9 | 5,952 | 0,8422 |  |
| sex | 1 | 8,157 | 8,157 | 1,154 |  |
| diet | 2 | 48,71 | 24,36 | 3,446 |  |
|  |  |  |  |  |  |
| Bonferroni posttests | | |  |  |  |
| male vs female |  |  |  |  |  |
| diet | Difference | t | P value | Summary |  |
| control | -0,8763 | 0,5629 | P > 0,05 | ns |  |
| HDD | -2,415 | 1,633 | P > 0,05 | ns |  |
| HFD | 0,4081 | 0,2535 | P > 0,05 | ns |  |

| Table Analyzed | HDL |  |  |  |  |
| --- | --- | --- | --- | --- | --- |
|  |  |  |  |  |  |
| Two-way ANOVA | |  |  |  |  |
|  |  |  |  |  |  |
| Source of Variation | % of total variation | P value |  |  |  |
| Interaction | 19,9 | 0,0147 |  |  |  |
| sex | 1,66 | 0,3776 |  |  |  |
| diet | 8,66 | 0,1397 |  |  |  |
|  |  |  |  |  |  |
| Source of Variation | P value summary | Significant? | |  |  |
| Interaction | * | Yes |  |  |  |
| sex | ns | No |  |  |  |
| diet | ns | No |  |  |  |
|  |  |  |  |  |  |
| Source of Variation | Df | Sum-of-squares | Mean square | F |  |
| Interaction | 2 | 669,3 | 334,7 | 4,792 |  |
| sex | 1 | 55,81 | 55,81 | 0,7992 |  |
| diet | 2 | 291,4 | 145,7 | 2,087 |  |
|  |  |  |  |  |  |
| Bonferroni posttests | | |  |  |  |
| male vs female |  |  |  |  |  |
| diet | Difference | t | P value | Summary |  |
| control | 7,059 | 1,564 | P > 0,05 | ns |  |
| HDD | -9,265 | 1,993 | P > 0,05 | ns |  |
| HFD | 9,335 | 2,008 | P > 0,05 | ns |  |

| Table Analyzed | Trigliceride | |  |  |  |
| --- | --- | --- | --- | --- | --- |
|  |  |  |  |  |  |
| Two-way ANOVA | |  |  |  |  |
|  |  |  |  |  |  |
| Source of Variation | % of total variation | P value |  |  |  |
| Interaction | 0,85 | 0,765 |  |  |  |
| sex | 18,36 | 0,0016 |  |  |  |
| diet | 22,72 | 0,0023 |  |  |  |
|  |  |  |  |  |  |
| Source of Variation | P value summary | Significant? | |  |  |
| Interaction | ns | No |  |  |  |
| sex | ** | Yes |  |  |  |
| diet | ** | Yes |  |  |  |
|  |  |  |  |  |  |
| Source of Variation | Df | Sum-of-squares | Mean square | F |  |
| Interaction | 2 | 1026 | 512,9 | 0,2698 |  |
| sex | 1 | 22157 | 22157 | 11,66 |  |
| diet | 2 | 27417 | 13709 | 7,213 |  |
|  |  |  |  |  |  |
| Bonferroni posttests | | |  |  |  |
| male vs female |  |  |  |  |  |
| diet | Difference | t | P value | Summary |  |
| control | -42,14 | 1,868 | P > 0,05 | ns |  |
| HDD | -58,86 | 2,526 | P < 0,05 | * |  |
| HFD | -35,37 | 1,518 | P > 0,05 | ns |  |

| Table Analyzed | ALT |  |  |  |  |
| --- | --- | --- | --- | --- | --- |
|  |  |  |  |  |  |
| Two-way ANOVA | |  |  |  |  |
|  |  |  |  |  |  |
| Source of Variation | % of total variation | P value |  |  |  |
| Interaction | 1,12 | 0,8888 |  |  |  |
| sex | 13,19 | 0,1123 |  |  |  |
| diet | 0,62 | 0,9371 |  |  |  |
|  |  |  |  |  |  |
| Source of Variation | P value summary | Significant? | |  |  |
| Interaction | ns | No |  |  |  |
| sex | ns | No |  |  |  |
| diet | ns | No |  |  |  |
|  |  |  |  |  |  |
| Source of Variation | Df | Sum-of-squares | Mean square | F |  |
| Interaction | 2 | 961,1 | 480,5 | 0,1187 |  |
| sex | 1 | 11286 | 11286 | 2,788 |  |
| diet | 2 | 528,2 | 264,1 | 0,06523 |  |
|  |  |  |  |  |  |
| Bonferroni posttests | | |  |  |  |
| male vs female |  |  |  |  |  |
| diet | Difference | t | P value | Summary |  |
| control | -34,08 | 0,8298 | P > 0,05 | ns |  |
| HDD | -64,33 | 1,324 | P > 0,05 | ns |  |
| HFD | -40,1 | 0,7532 | P > 0,05 | ns |  |

| Table Analyzed | AST |  |  |  |  |
| --- | --- | --- | --- | --- | --- |
|  |  |  |  |  |  |
| Two-way ANOVA | |  |  |  |  |
|  |  |  |  |  |  |
| Source of Variation | % of total variation | P value |  |  |  |
| Interaction | 29,98 | 0,0257 |  |  |  |
| sex | 3,64 | 0,3123 |  |  |  |
| diet | 6,15 | 0,4199 |  |  |  |
|  |  |  |  |  |  |
| Source of Variation | P value summary | Significant? | |  |  |
| Interaction | * | Yes |  |  |  |
| sex | ns | No |  |  |  |
| diet | ns | No |  |  |  |
|  |  |  |  |  |  |
| Source of Variation | Df | Sum-of-squares | Mean square | F |  |
| Interaction | 2 | 24965 | 12482 | 4,42 |  |
| sex | 1 | 3035 | 3035 | 1,075 |  |
| diet | 2 | 5120 | 2560 | 0,9066 |  |
|  |  |  |  |  |  |
| Bonferroni posttests | | |  |  |  |
| male vs female |  |  |  |  |  |
| diet | Difference | t | P value | Summary |  |
| control | -30,71 | 0,9221 | P > 0,05 | ns |  |
| HDD | -23,33 | 0,6012 | P > 0,05 | ns |  |
| HFD | 124,3 | 2,796 | P < 0,05 | * |  |

| Table Analyzed | pcsk9 |  |  |  |  |
| --- | --- | --- | --- | --- | --- |
|  |  |  |  |  |  |
| Two-way ANOVA | |  |  |  |  |
|  |  |  |  |  |  |
| Source of Variation | % of total variation | P value |  |  |  |
| Interaction | 1,8 | 0,6113 |  |  |  |
| sex | 1,93 | 0,3083 |  |  |  |
| diet | 39,24 | 0,0003 |  |  |  |
|  |  |  |  |  |  |
| Source of Variation | P value summary | Significant? | |  |  |
| Interaction | ns | No |  |  |  |
| sex | ns | No |  |  |  |
| diet | *** | Yes |  |  |  |
|  |  |  |  |  |  |
| Source of Variation | Df | Sum-of-squares | Mean square | F |  |
| Interaction | 2 | 1817 | 908,7 | 0,5 |  |
| sex | 1 | 1950 | 1950 | 1,073 |  |
| diet | 2 | 39559 | 19779 | 10,88 |  |
|  |  |  |  |  |  |
| Bonferroni posttests | | |  |  |  |
| male vs female |  |  |  |  |  |
| diet | Difference | t | P value | Summary |  |
| control | -31,03 | 1,308 | P > 0,05 | ns |  |
| HDD | -15,3 | 0,5929 | P > 0,05 | ns |  |
| HFD | 2,493 | 0,1051 | P > 0,05 | ns |  |

| Table Analyzed | glucose |  |  |  |  |
| --- | --- | --- | --- | --- | --- |
|  |  |  |  |  |  |
| Two-way ANOVA | |  |  |  |  |
|  |  |  |  |  |  |
| Source of Variation | % of total variation | P value |  |  |  |
| Interaction | 10,4 | 0,064 |  |  |  |
| sex | 2,62 | 0,2289 |  |  |  |
| diet | 26,64 | 0,0018 |  |  |  |
|  |  |  |  |  |  |
| Source of Variation | P value summary | Significant? | |  |  |
| Interaction | ns | No |  |  |  |
| sex | ns | No |  |  |  |
| diet | ** | Yes |  |  |  |
|  |  |  |  |  |  |
| Source of Variation | Df | Sum-of-squares | Mean square | F |  |
| Interaction | 2 | 6012 | 3006 | 2,977 |  |
| sex | 1 | 1514 | 1514 | 1,499 |  |
| diet | 2 | 15395 | 7697 | 7,624 |  |
|  |  |  |  |  |  |
| Bonferroni posttests | |  |  |  |  |
| male vs female | |  |  |  |  |
| diet | Difference | t | P value | Summary |  |
| control | -18,25 | 1,11 | P > 0,05 | ns |  |
| HDD | 14,57 | 0,8243 | P > 0,05 | ns |  |
| HFD | 40,32 | 2,281 | P > 0,05 | ns |  |

| Table Analyzed | creatinine clearance | | |  |  |
| --- | --- | --- | --- | --- | --- |
|  |  |  |  |  |  |
| Two-way ANOVA | |  |  |  |  |
|  |  |  |  |  |  |
| Source of Variation | % of total variation | P value |  |  |  |
| Interaction | 1,98 | 0,4187 |  |  |  |
| sex | 45,34 | < 0,0001 |  |  |  |
| diet | 9,28 | 0,0229 |  |  |  |
|  |  |  |  |  |  |
| Source of Variation | P value summary | Significant? | |  |  |
| Interaction | ns | No |  |  |  |
| sex | *** | Yes |  |  |  |
| diet | * | Yes |  |  |  |
|  |  |  |  |  |  |
| Source of Variation | Df | Sum-of-squares | Mean square | F |  |
| Interaction | 2 | 0,1451 | 0,07253 | 0,8907 |  |
| sex | 1 | 3,322 | 3,322 | 40,79 |  |
| diet | 2 | 0,6801 | 0,3401 | 4,176 |  |
|  |  |  |  |  |  |
| Bonferroni posttests | | |  |  |  |
| male vs female | |  |  |  |  |
| diet | Difference | t | P value | Summary |  |
| control | -0,6796 | 4,763 | P<0,001 | *** |  |
| HDD | -0,4018 | 2,634 | P < 0,05 | * |  |
| HFD | -0,5705 | 3,74 | P<0,01 | ** |  |

Supplementary Figures 1 and 2.

Controls HDD HFD

Controls HDD HFD

* *P*<0.05. vs. controls; ## *P*<0.01 HDD vs. HFD Means ± SE are presented

Supplementary Figure 3. Uncropped blots


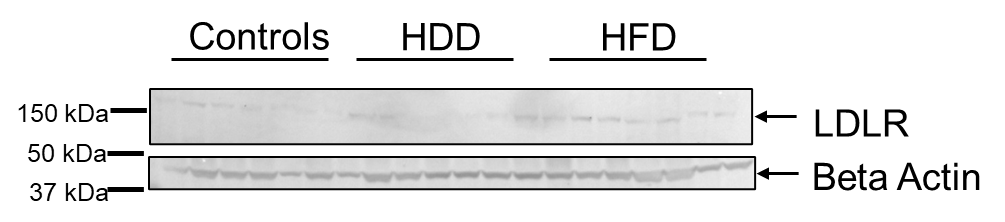

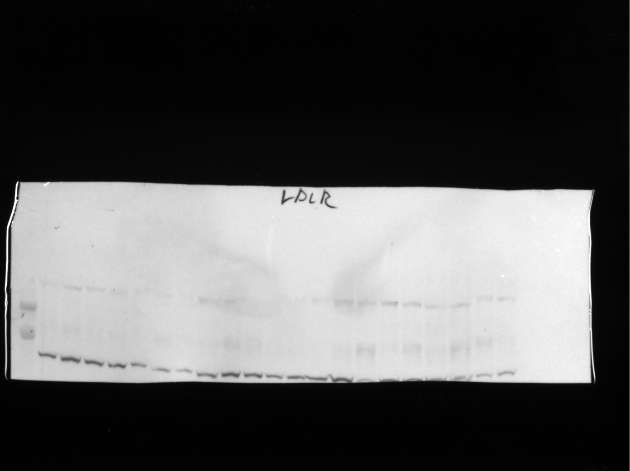


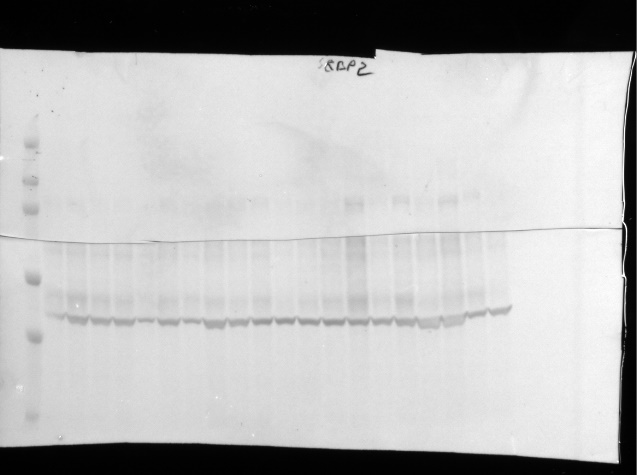


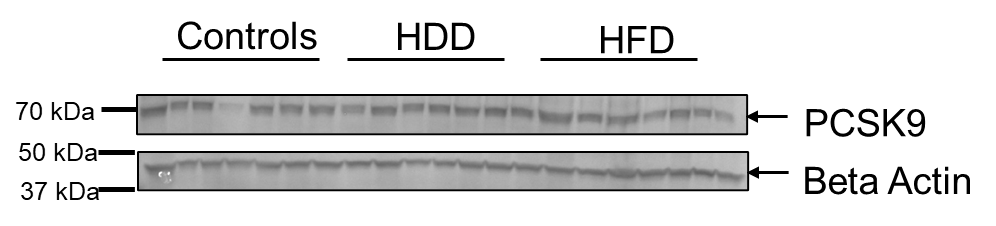

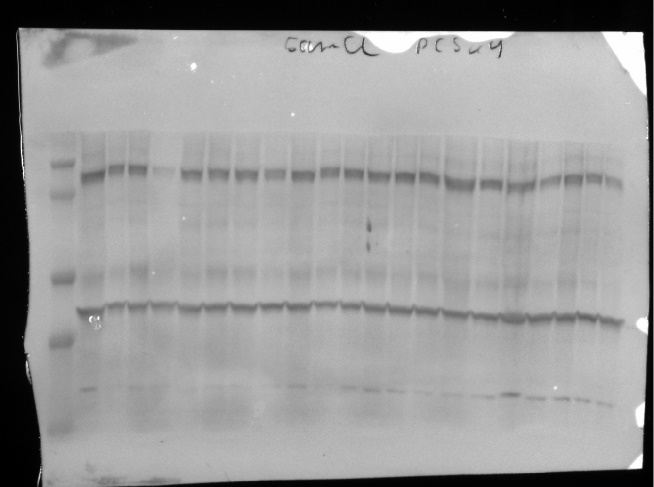

Supplement: Supplementary file 4 — Supplementary Material 4 [file 41598_2024_74193_MOESM4_ESM.docx]
